# Identification and characterization of ncRNA-associated ceRNA networks in *Arabidopsis* leaf development

**DOI:** 10.1186/s12864-018-4993-2

**Published:** 2018-08-13

**Authors:** Xianwen Meng, Peijing Zhang, Qi Chen, Jingjing Wang, Ming Chen

**Affiliations:** 1 Department of Bioinformatics, the State Key Laboratory of Plant Physiology and Biochemistry, Institute of Plant Science, College of Life Sciences, Zhejiang University, 866 Yuhangtang Road, Hangzhou, 310058 Zhejiang Province China; 2 State Key Laboratory of Crop Biology, College of Agronomy, Shandong Agricultural University, 61 Daizong Road,, Tai’an, 271018 Shandong Province China

**Keywords:** Leaf development, Noncoding RNAs, circRNAs, lncRNAs, ceRNA

## Abstract

**Background:**

Leaf development is a complex biological process that is accompanied by wide transcriptional changes. Many protein-coding genes have been characterized in plant leaves, but little attention has been given to noncoding RNAs (ncRNAs). Moreover, increasing evidence indicates that an intricate interplay among RNA species, including protein-coding RNAs and ncRNAs, exists in eukaryotic transcriptomes, however, it remains elusive in plant leaves.

**Results:**

We detected novel ncRNAs, such as circular RNAs (circRNAs) and long noncoding RNAs (lncRNAs), and further constructed and analyzed their associated competitive endogenous RNA (ceRNA) networks in *Arabidopsis* leaves. Transcriptome profiling showed extensive changes during leaf development. In addition, comprehensive detection of circRNAs in other plant leaves suggested that circRNAs are widespread in plant leaves. To investigate the complex post-transcriptional interactions in *Arabidopsis* leaves, we constructed a global circRNA/lncRNA-associated ceRNA network. Functional analysis revealed that ceRNAs were highly correlated with leaf development. These ceRNAs could be divided into six clusters, which were enriched for different functional classes. Stage-specific ceRNA networks were further constructed and comparative analysis revealed different roles of stage common and specific hub ceRNAs.

**Conclusions:**

Our results demonstrate that understanding the ceRNA interactions will lead insights into gene regulations implicated in leaf development.

**Electronic supplementary material:**

The online version of this article (10.1186/s12864-018-4993-2) contains supplementary material, which is available to authorized users.

## Background

The leaf is an important organ that intercepts light and exchange gasses. Plant leaves undergo developmental and physiological changes during their lifespans. A leaf develops into a mature photosynthetic organ during the growth stage, and undergoes organic-level senescence during the final stage of leaf development. Senescence involves the reallocation of nutrients from the leaves to seeds or fruits. This process can be naturally induced or stimulated by external factors [1]. Premature senescence will cause reduced yield and quality of crops. Thus, an improved understanding of leaf development is essential for increasing plant growth and productivity. Leaf development is characterized by altered metabolic and signaling pathways, and accompanied by extensive changes in the transcriptome [2, 3]. However, the underlying molecular regulatory mechanisms are largely unclear.

In plants, tissue development is a tightly regulated process. Identifying the regulatory mechanisms that govern this process is of great interest to biologists. MicroRNAs (miRNAs), a class of small ncRNAs, have been studied widely since they are implicated in post-transcriptional RNA silencing. Some transcripts, named “miRNA sponges”, could efficiently inhibit miRNA functions by sequestering them. Particularly, the first endogenous miRNA sponge was discovered in plants [4]. Therefore, construction of miRNA-target interactions is a necessary way toward exploring the roles they play in plants. In addition, competing endogenous RNAs have emerged as a novel type of miRNA-mediated gene regulation [5]. CeRNAs are transcripts that share miRNA binding sites, thereby they communicate with and co-regulate each other by competing for miRNA binding. In recent years, numerous studies have demonstrated that ceRNAs may represent a widespread layer of gene regulation involved in human diseases and tissue development [6, 7]. Although ceRNAs are widespread in plant species [8], there is little knowledge about their functional roles in plants.

Circular RNAs constitute a family of transcripts with covalently closed structure. Recent studies have revealed that several circRNAs could be translated in human, but most circRNAs belong to ncRNAs since they are not associated with polysomes [9–11], therefore, unlike protein-coding RNAs, circRNAs with no protein-coding potential may function in cells directly just like other ncRNAs. Increasing evidence suggests that circRNAs are potential regulators in RNA world and aberrant expression of circRNAs correlates with human diseases, especially human cancers [12, 13]. Long noncoding RNA is another type of widespread and endogenous ncRNA. Studies have shown that some circRNAs and lncRNAs, harboring multiple binding sites for miRNAs, could regulate the activity of miRNAs by sponging miRNAs [14–16]. Thus, they are potential ceRNAs that sequester miRNAs to suppress their function [5, 17]. Recent studies have shed light on the roles of interactions between ncRNAs and protein-coding mRNAs in human cancers [18], but the properties and dynamics of ncRNA associated interactions in plants remain elusive.

To better understand the dynamic processes and global control of *Arabidopsis* leaf development, we aimed to identify the circRNAs and lncRNAs using high-throughput sequencing data from developing leaves. The other objectives of our work were to characterize diverse transcripts, explore the conservation of circRNAs in plants, construct ncRNA-associated ceRNA networks and conduct a network analysis.

## Methods

### Data collection

We collected smRNA-Seq and rRNA depleted total RNA-Seq data of *Arabidopsis* leaves from NCBI GEO database (http://www.ncbi.nlm.nih.gov/; GSE43616). Leaves were sampled at 2d intervals from the ages of 4 to 30d, including the entire lifespan of a leaf. Total RNA-Seq data of other plant leaves were also from GEO database (*Triticum aestivum*, GSE58805; *Glycine max*, GSE69469; *Zea mays*, GSE71046; *Oryza sativa Indica*, GSE74465). Genes involved in *Arabidopsis* leaf development or senescence were from LEAFDATA [19] and LSD [20]. *Arabidopsis* transcription factors (TFs) were downloaded from AtTFDB [21] and PlantTFDB [22].

### Computational identification of circRNAs and lncRNAs

To detect most back-splice junction sites of circRNAs in *Arabidopsis* leaves, we used three tools, find_circ [14], circRNA_finder [23] and CIRI2 [24] since the combination of their results could achieve more unbiased circRNA detection [25]. If the back-splice site was supported by at least two junction reads, then, it was selected as a circRNA candidate.

To predict novel lncRNAs in *Arabidopsis* leaves, the sequencing reads were first aligned to *Arabidopsis* genome using Tophat2 [26]. Then, the transcripts were assembled using Cufflinks [27] and the assembled transcripts from different samples were merged together using Cuffmerge. The transcripts annotated with class code of ‘i’, ‘x’ or ‘u’ were selected if they were generated from noncoding or intergenic regions. The protein-coding potential of these selected transcripts was then calculated by CNCI [28]. Finally, the transcripts with no protein-coding potential, at least 200 nucleotides long and limited ORF length (no longer than 120 amino acids) were filtered as novel lncRNA candidates. In addition, lncRNAs annotated in TAIR10 (http://www.arabidopsis.org/) and experimentally identified lncRNAs from PLNlncRbase [29] were also included in our study.

### Transcript quantification and differential expression analysis

The fragments per kilobase of transcript per million mapped reads (FPKM) was first calculated and then log2 transformed after adding 1 to quantify the mRNA or lncRNA expression levels. To filter the transcripts exhibiting reproducible temporal expression levels, the Pearson correlation coefficient for each transcript was calculated, whose cutoff was set to > 0.5 and corresponding *P*-value was set to < 0.05. CircRNA expression, measured as reads per million (RPM), was normalized by dividing the junction read counts to the total number of sequencing reads in the each sample. For miRNAs, the read counts were first calculated by miRExpress [30] and further RPM-normalized. Similarly, the RPM-normalized expression value was then log2 transformed. The transcripts with low expression levels throughout the leaf lifespan were filtered out (circRNA counts > = 2; lncRNA and mRNA FPKM > = 1; miRNA RPM > = 1; at least one sample).

A t-test was used to identify the differentially expressed transcripts between different development stages. In this case, the transcripts with *P*-value < 0.05 were considered as significantly expressed transcripts.

### MiRNA target prediction

Known mature miRNA sequences for *Arabidopsis* were downloaded from miRBase (release 21) [31]. Together with mRNAs, circRNAs and lncRNAs identified above were used as the target prediction library. TargetFinder [32] and psRNATarget [33] were used with the default parameters to identify miRNA target sites, and the prediction results were combined to achieve high true positive coverage [34].

### Construction of ceRNA networks related to the development of *Arabidopsis* leaves

After obtaining the miRNA-mRNA, miRNA-circRNA and miRNA-lncRNA regulatory data, we employed the following principles to identify ceRNA pairs in different development stages. Firstly, a ceRNA pair should be significantly regulated by common expressed miRNAs during leaf development. Therefore, a hypergeometric test was used to measure the significance of shared miRNAs. We considered *P*-value < 0.05 was statistically significant. Secondly, the Pearson correlation coefficient (R) of each candidate ceRNA pair was computed. All the candidate ceRNA pairs with *R* > 0.5 and *P*-value < 0.05 were selected as ceRNA interactions. Finally, we assembled all the identified ceRNA pairs, generating the circRNA/lncRNA-associated ceRNA networks in developing leaves.

### Functional enrichment analysis

Functional enrichment analysis at the Gene Ontology (GO) level was performed using agriGO [35]. The biological function categories with false discovery rate (FDR) adjusted P-value < 0.05 were statistically significant. In addition, the semantic similarity among GO terms was calculated using GOSemSim package [36].

## Results

### Expression patterns of ncRNAs in *Arabidopsis* leaves

**Fig. 1:**
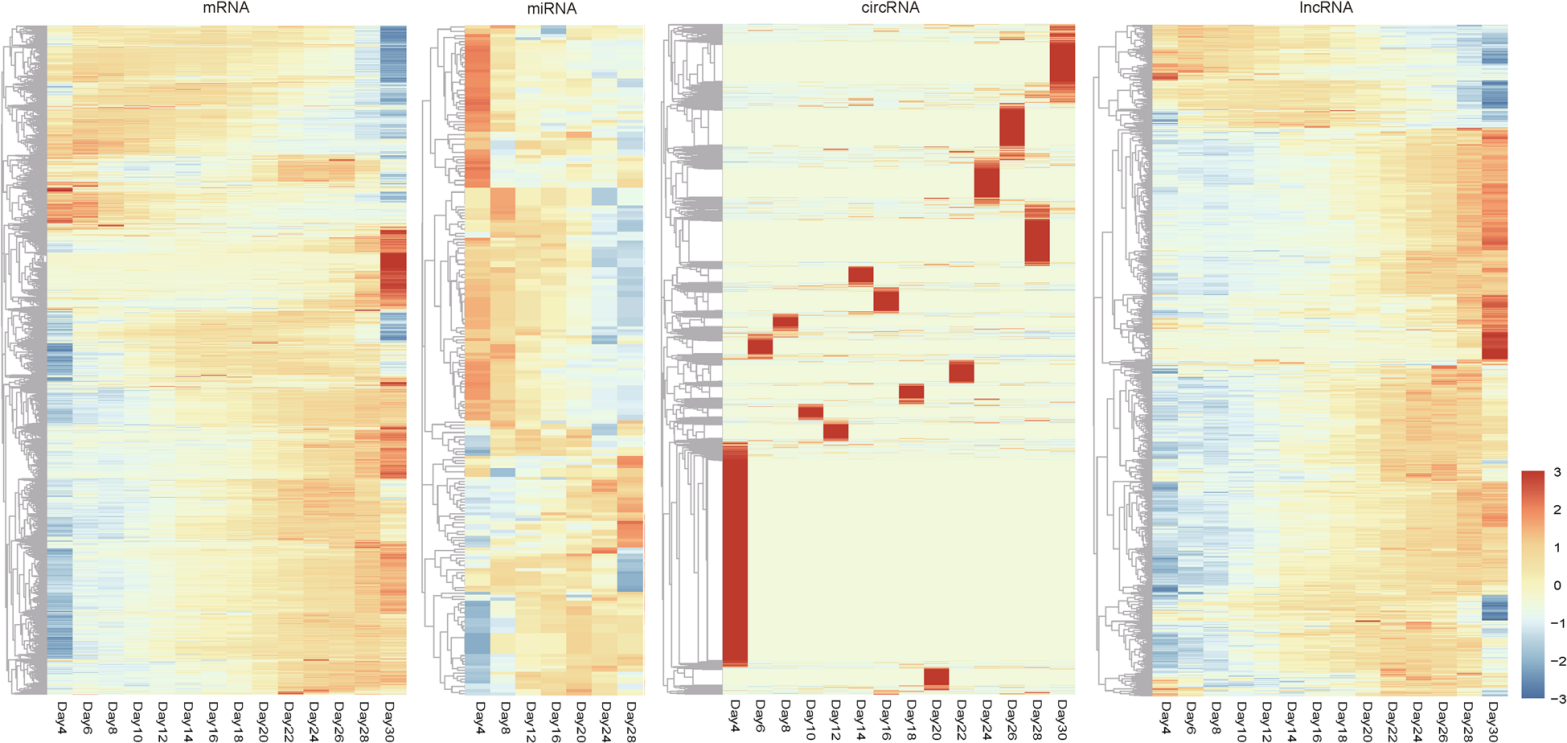
Transcriptome profiles of *Arabidopsis* leaves

Based on backspliced reads in rRNA depleted RNA-Seq data, we characterized 11,490 circRNAs, of which 9771 (85.04%) were exonic circRNAs (ecircRNAs) generated from exons of a single protein-coding gene. The remaining circRNAs included 47 intronic circRNAs, 1316 intergenic circRNAs, and 356 “other” circRNAs that were generated from two or more different protein-coding genes. These results indicated that circRNAs in *Arabidopsis* leaves were mainly generated from coding regions. Unlike lncRNAs or miRNAs, circRNAs tend to be time point-specific expressed (Fig. 1). A recent study indicated that some plant circRNAs exhibited significant co-expression with their parent genes [37]. To investigate the expression relationship between ecircRNAs and their linear counterparts in *Arabidopsis* leaves, we calculated the Pearson correlation coefficients (R) based on their expression levels. Only 7.68% of the ecircRNAs showed a significant co-expression with their parent genes (*R* > 0.5 and *P*-value < 0.05), suggesting that the splicing machinery involved in ecircRNA biogenesis in *Arabidopsis* leaves might be different from canonical splicing. Next, we assessed the relationship between circularizing genes and leaf development. Surprisingly, genes that generated circRNAs were found to be biased for leaf developmental genes (P-value = 0, hypergeometric test), and functional enrichment analysis revealed that parent genes of top100 expressed circRNAs were enriched in crucial biological processes in plant leaves, such as photosynthesis, stress response processes and other metabolic pathways (Additional file 1: Table S1), indicating these RNA circles may not only correlate with leaf development and senescence, but also function in many important biological processes in *Arabidopsis* leaves.

746 lncRNAs were detected from the RNA-Seq data of *Arabidopsis* leaves. The lncRNAs from public databases were also included. Finally, 795 lncRNAs were expressed during leaf development. Meanwhile, 227 miRNAs were found to be expressed in *Arabidopsis* leaves. Expression patterns of mRNAs, lncRNAs and miRNAs exhibited that there was a dramatic divergence between the early and late stages of leaf development, and most transcripts tended to be differentially expressed between different stages (Fig. 1).

### Conservation of circRNAs in plant leaves

To investigate whether circRNAs also exist in other plant leaves, we further analyzed the public sequencing data and identified 473, 36,910, 3854, 1322 circRNAs in *Glycine max*, *Oryza sativa*, *Triticum aestivum*, *Zea mays*, respectively (Additional file 2: Table S2). Among these RNA circles, most were exonic or intergenic circRNAs. BLAST search analysis revealed that the parent genes generating ecircRNAs exhibited some conservation (Additional file 3: Table S3). For example, in *Arabidopsis* and *Oryza sativa*, 1797 parent genes were orthologous, accounting for 47.46% and 26.28% parent genes, respectively. Particularly, 13 orthologous parent genes were shared by these five species and GO enrichment analysis revealed that these genes were highly correlated with photosynthesis (GO:0009765, FDR = 3.90 × 10^−18^), suggesting that the circRNAs generating from these orthologous genes might also be involved in photosynthesis in plant leaves. In addition, to facilitate plant researchers to explore our findings, we further constructed a database, LeafcircBase (http://bis.zju.edu.cn/LeafcircBase/), the first database focusing on circRNAs in plant leaves. It provided the information for genomic location and conservation of circRNAs, thus benefiting the study of the roles of circRNAs in plant leaves.

### CircRNA/lncRNA-associated ceRNA network in *Arabidopsis* leaves

**Fig. 2:**
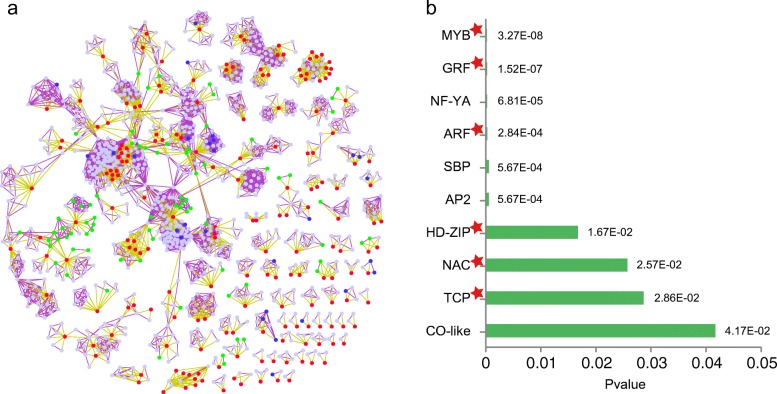
Leaf-associated TF family enriched ceRNA network. **a** Global ceRNA network in the leaf lifespan. The red, light blue, green, blue nodes represent miRNAs, mRNAs, circRNAs and lncRNAs, respectively. Yellow edges represent miRNA-target interactions while purple edges represent the competing relationships. **b** Ten TF families were enriched in the ceRNA network. Particularly, six TF families marked with a red star have been reported to be implicated in leaf development

To evaluate the ncRNA-associated ceRNA interaction landscape involved in *Arabidopsis* leaves, we constructed the ceRNA network using a multi-step approach, where the similarity of the miRNA regulatory patterns and the similarity in expression were both considered. First, the miRNA-binding sites in transcripts were identified. In total, we obtained 5115 miRNA-mRNA, 1045 miRNA-circRNA and 149 miRNA-lncRNA interactions. Then, RNA pairs sharing a significant number of miRNAs were identified. Finally, we required that the filtered RNA pairs should be co-expressed during the entire lifespan. There were 4535 ceRNA pairs and 1782 miRNA-target interactions in the global ceRNA network and it tended to form discrete clusters (Fig. 2a). Particularly, 75 circRNAs and 28 lncRNAs were implicated in the network. Understanding leaf development in the context of ceRNA network allows us to explore the functions of circRNAs and lncRNAs.

To illustrate that ceRNA network is a potential regulatory mechanism involved in leaf development, we conducted a functional enrichment analysis based on the GO annotation. We found the protein-coding genes in the network were highly correlated with developmental processes (Additional file 4: Table S4). Particularly, the leaf-associated biological processes, such as leaf development (GO:0048366, FDR = 6.8 × 10^−7^) and leaf morphogenesis (GO:0009965, FDR = 3.3 × 10^−4^), were significantly enriched. TFs, acting as an important type of regulators, are involved in leaf growth and senescence [38]. We found 10 TF families were significantly enriched in the ceRNA network (Fig. 2b). Notably, six of them were implicated in *Arabidopsis* growth or senescence [39–44]. Together, these ceRNAs were highly correlated with leaf development, thus, they can be used to explain the mechanisms of the developmental process or discover novel developmental genes.

### CeRNAs exhibit dynamic expression during leaf development

**Fig. 3:**
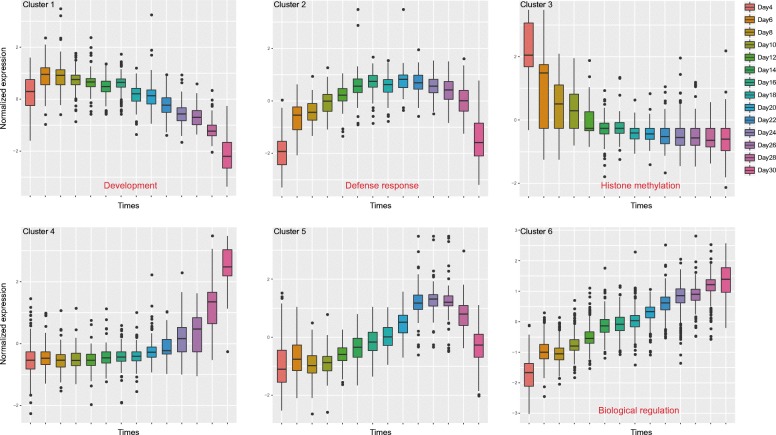
CeRNA clusters with different expression patterns. Clustering analysis was performed on the time point data for transcripts in the ceRNA network. Six clusters were obtained. Selected enriched GO terms are indicated

Next, we profiled the expressions of ceRNAs to investigate the global expression pattern. Clustering analysis using k-means method was performed to identify ceRNA clusters. Finally, six clusters were obtained. Most ceRNAs showed dynamic expression, indicating the temporally related expression of them during leaf development (Fig. 3). In addition, we found different ceRNA clusters tend to be associated with different cellular processes. For example, the ceRNA cluster showing high expression level during the early growth stage and decreasing expression level during the late senescence stage (cluster 1) was significantly enriched for genes linked to developmental processes. Particularly, 6 circRNAs and 2 lncRNAs were included in this cluster, indicating these ncRNAs might also be involved in the leaf development (Additional file 5: Table S5). Various biotic and abiotic stresses are implicated in the process of leaf development, especially during leaf senescence. The genes associated with defense response exhibited an increasing expression level during the early stage but a decreasing expression level during the late stage (cluster 2). Moreover, the ceRNAs with increasing expression level during the entire lifespan (cluster 6) were involved in biological regulation processes, suggesting that the strength of regulatory systems is changing during leaf development. Together, there was a dramatic difference in expression of ceRNAs between the early and late stages during leaf development, reflecting the dynamic nature and flexibility of leaf development.

We further clustered the samples throughout the leaf lifespan. Surprisingly, the samples at Day16 and Day18, which were ambiguous at the level of phenotypic classification [45], were clustered into the first group (Additional file 6: Figure S1a). Similarly, expression profiling of leaf-associated genes exhibited the same clustering result (Additional file 6: Figure S1b). Accordingly, the leaf lifespan were divided into two main stages: growth stage (Day4–18) and senescence stage (Day20–30) based on the expression pattern of ceRNAs and leaf-associated genes. Notably, 13,960 (44.2%) transcripts were differentially expressed between these two stages, reflecting the divergence between the molecular bases of the two stages. These differentially expressed transcripts serve as an important resource for understanding transcriptional programs. The dynamic and stage-specific expression of transcripts indicates that they are potential regulatory molecules in leaf growth and senescence in *Arabidopsis*.

### Different roles of stage common and specific hub ceRNAs

**Fig. 4:**
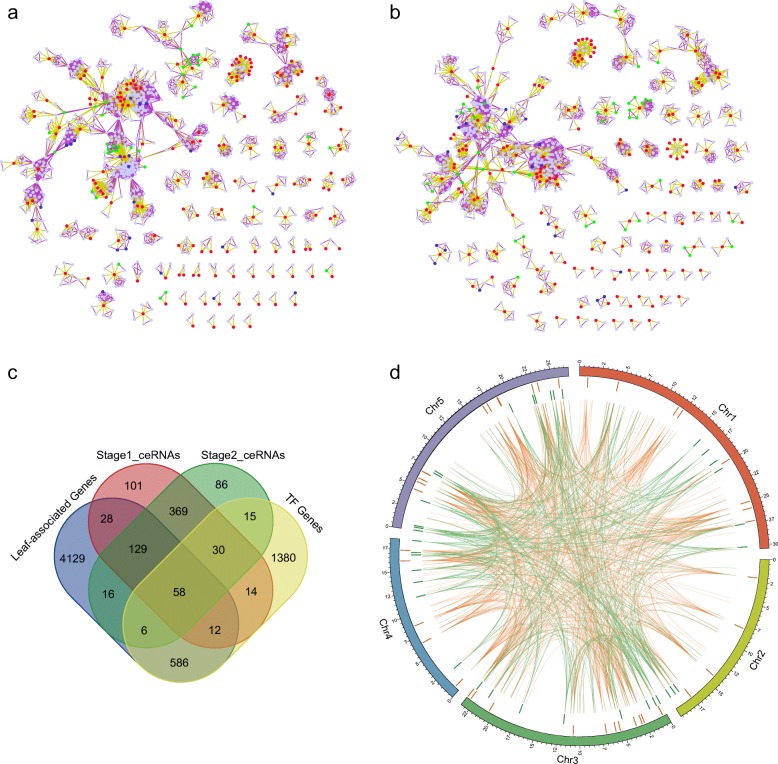
Stage specific ceRNA networks. Similarly, the ceRNA networks in (**a**) growth stage and (**b**) senescence stage tend to form discrete clusters. **c** The stage specific ceRNAs were enriched for leaf-associated genes and TF genes. **d** Circos plot for stage hub interactions. The orange links represent hub interactions in growth stage while the green links represent hub interactions in senescence stage. The tracks show the distributions of hub ceRNAs in chromosomes

Since the lifespan of leaves could be divided into two main stages, we further constructed the ceRNA networks in each stage (Fig. 4a, b). Then, we compared the ceRNAs with *Arabidopsis* TFs, as well as previously identified leaf-associated genes (Fig. 4c). As expected, the genes in ceRNA networks are enriched for TF genes and leaf-associated genes (*P*-values < 0.001, randomization test).

Hub nodes with high connectivity are important to a network. In our analysis, the hubs were defined as the top 10% of the nodes with highest degree. There were 43 common hub ceRNAs between the ceRNA networks of the two stages. To understand the roles of common hub ceRNAs in leaves, we extracted the ceRNA interactions among the common hub ceRNAs and conducted a functional enrichment analysis. The result indicated that the common hub ceRNAs were associated with basic biological functions, such as amino acid and carbohydrate metabolic processes (Additional file 7: Table S6). In addition, the common hub ceRNAs were regulated by 21 miRNAs, ten of which belong to miR156 family. Notably, miR156 is essential to vegetative phase change in *Arabidopsis* [46].

To investigate the developmental mechanisms of different stages, we further focused on the stage specific hub ceRNAs. There were 41 and 39 specific hub ceRNAs within the ceRNA networks of growth stage and senescence stage, respectively. Similarly, we extracted their ceRNA interactions in each stage (Fig. 4d). Functional analysis revealed that hub interactions in the early stage were highly correlated with developmental, biosynthetic and metabolic processes, as well as positive regulations of these processes (Additional file 8: Figure S2a). However, as for the hub interactions in the late stage, they were implicated in less developmental, biosynthetic and metabolic processes, indicating life events in late stage were less active (Additional file 8: Figure S2b). Moreover, chloroplast organization, thylakoid membrane organization and plastid membrane organization were assigned to the senescence stage, suggesting that these ceRNAs might be responsible for the changes of chloroplast and leaf phenotype, causing leaf senescence.

**Fig. 5:**
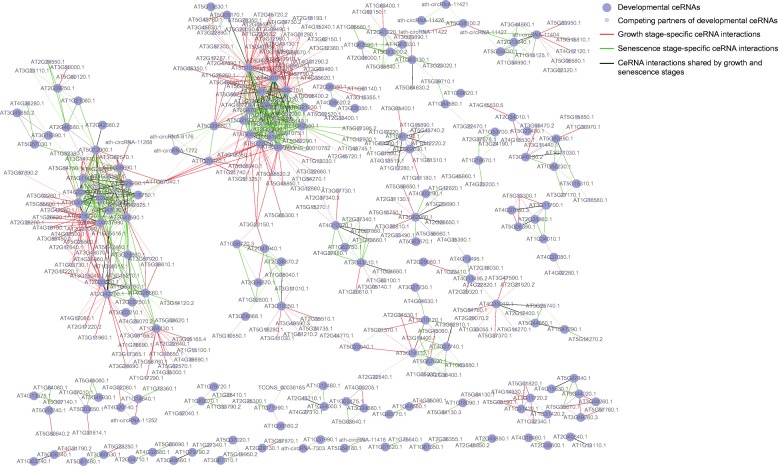
Developmental gene competing interactions between growth and senescence stages

The difference of developmental gene competing interactions between growth and senescence stages was further explored. We focused on ceRNAs from cluster 1 identified from global ceRNA network since they tend to be associated with developmental processes. The competing interactions of these developmental genes change dramatically (Fig. 5), implying the discordance of molecular interactions between growth and senescence stages.

Taken together, these results indicated that exploring the properties of ceRNA networks could help investigate the genes contributing to leaf stage development.

## Discussion

The application of recent genomics technology has enabled the identification of leaf-associated genes. A large number of genes have been found to exhibit a differential expression profile. However, the strategies that these genes utilize to regulate leaf development have not been well characterized. Thus, we presented a ceRNA network analysis to elucidate the potential regulatory mechanisms during leaf development.

Recently, increasing studies indicate that ncRNAs play important roles during plant development. Using high-throughput sequencing data, we identified novel noncoding transcripts, including circRNAs and lncRNAs. 11,490 circRNAs and 746 lncRNAs were expressed in *Arabidopsis* leaves, implying that ncRNAs are abundant in developing leaves. Our study revealed the first prediction of circRNA and lncRNA expression patterns in the context of *Arabidopsis* leaf development. The transcriptome profiles indicated that an extensive change occurs during leaf development, which is concordant with previous findings. Many ncRNAs exhibited differential expression between growth and senescence stages. Furthermore, we detected the circRNAs in other four model plant leaves, providing a unique leaf circRNA resource. The linear parent genes of ecircRNAs showed some conservation and they tend to be implicated in photosynthesis, indicating that ecircRNAs might be potential regulators in biological processes.

Constructing ceRNA networks is a novel strategy to explore gene functions, which is widely used in the study of human diseases. However, the competing relationships among coding and noncoding RNAs are rarely characterized in plants. In this study, we constructed a global circRNA/lncRNA-associated ceRNA network in developing leaves. Functional analysis revealed that these ceRNAs were significantly enriched for developmental genes. In addition, several leaf-associated TF families were enriched in the ceRNA network. Together, these results indicated that the competing interaction is a potential regulatory mechanism of leaf development and the ncRNAs involved in the ceRNA network might be novel regulators of leaf development.

Profiling the expressions of ceRNAs suggested a dynamic change during leaf development. CeRNA clusters with similar expression patterns were identified. Different clusters were significantly enriched for different biological processes, implying multiple regulatory or metabolic pathways were involved during leaf development. Particularly, functional enrichment analysis revealed that the mRNAs in cluster 1 were highly correlated with developmental processes. Except for mRNAs, 6 circRNAs and 2 lncRNAs were included in this cluster. Among these circRNAs, circRNA-11,426, circRNA-11,404, circRNA-11,421 were originated from the chloroplast genome, suggesting they might be involved in leaf development through regulating function of chloroplast, such as photosynthesis. We further constructed the ceRNA networks in growth and senescence stages. Network comparison revealed that common hub ceRNAs were associated with basic biological processes, while stage-specific hub ceRNAs were highly correlated with developmental processes that contributing the phenotype of a leaf.

## Conclusions

In this study, we identified the circRNAs and lncRNAs using high-throughput sequencing data from developing leaves and further constructed their associated ceRNA networks. Transcriptome profiles suggested a dramatic divergence between the early and late stages of leaf development. Comprehensive detection of circRNAs in other plant leaves formed a unique leaf circRNA resource. Sequence analysis revealed that circRNA parent genes showed some conservation. CeRNAs exhibited a dynamic expression during leaf development and clustering results suggested that the leaf lifespan could be divided into two main stages. Furthermore, functional roles of hub ceRNAs in the stage-specific ceRNA networks were characterized. Developmental gene competing interactions changed greatly between stages. Genome-wide identification of novel ncRNAs and construction of their associated ceRNA networks in *Arabidopsis* leaves could provide insights into the mechanisms of leaf development and identify potential regulators. Our results broaden the study of ncRNAs in plants, highlighting a regulatory role of ncRNA-associated ceRNA interactions in leaf development.

## Additional files

Additional file 1: **Table S1.** Functional enrichment analysis of ecircRNA parent genes. (XLSX 12 kb)

Additional file 2: **Table S2.** Statistics of circRNAs in plant leaves. (XLSX 9 kb)

Additional file 3: **Table S3.** Number of conserved circRNA parent genes. (XLSX 9 kb)

Additional file 4: **Table S4.** Functional enrichment analysis of ceRNAs. (XLSX 17 kb)

Additional file 5: **Table S5.** CeRNA clusters in global ceRNA network. (XLSX 33 kb)

Additional file 6: **Figure S1.** Clustering results of leave samples based on ceRNA and leaf-associated gene expressions. (PDF 2720 kb)

Additional file 7: **Table S6.** Functional enrichment analysis of common hub ceRNAs. (XLSX 10 kb)

Additional file 8: **Figure S2.** Enriched GO term interactive graph of hub ceRNA interactions. (PDF 472 kb)

## Abbreviations

ceRNA: Competitive endogenous RNA
circRNA: Circular RNA
ecircRNA: Exonic circular RNA
FDR: False discovery rate
FPKM: Fragment per kilobase of transcript per million mapped reads
GO: Gene ontology
lncRNA: Long noncoding RNA
miRNA: Microrna
ncRNA: Noncoding RNA
RPM: Reads per million
