# Supplementary figures and images for: Identification and characterization of ncRNA-associated ceRNA networks in Arabidopsis leaf development
Source: BMC Genomics. 2018 Aug 13;19:607. doi: 10.1186/s12864-018-4993-2 (PMC6090674; doi:10.1186/s12864-018-4993-2)

a

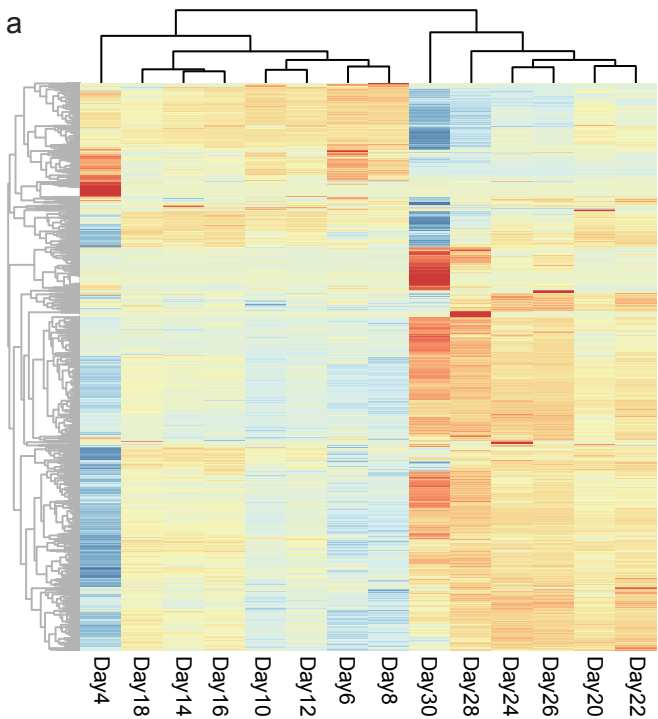

b

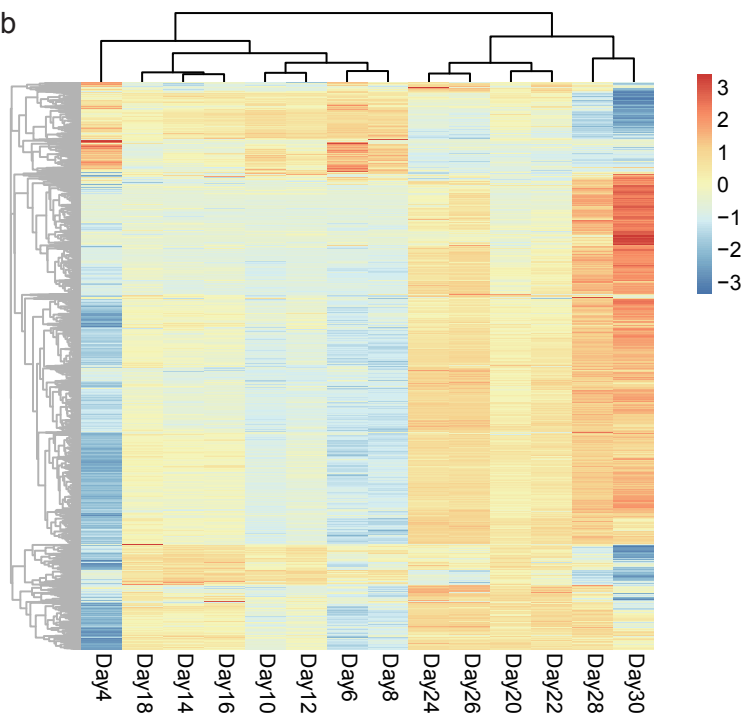

Supplement: Supplementary file 6 — Figure S1. Clustering results of leave samples based on ceRNA and leaf-associated gene expressions. (PDF 2720 kb) [file 12864_2018_4993_MOESM6_ESM.pdf]
