# Supplementary material for: Identification and characterization of ncRNA-associated ceRNA networks in Arabidopsis leaf development
Source: BMC Genomics. 2018 Aug 13;19:607. doi: 10.1186/s12864-018-4993-2 (PMC6090674; doi:10.1186/s12864-018-4993-2)
Supplement: Supplementary file 8 — Figure S2. Enriched GO term interactive graph of hub ceRNA interactions. (PDF 472 kb) [file 12864_2018_4993_MOESM8_ESM.pdf]

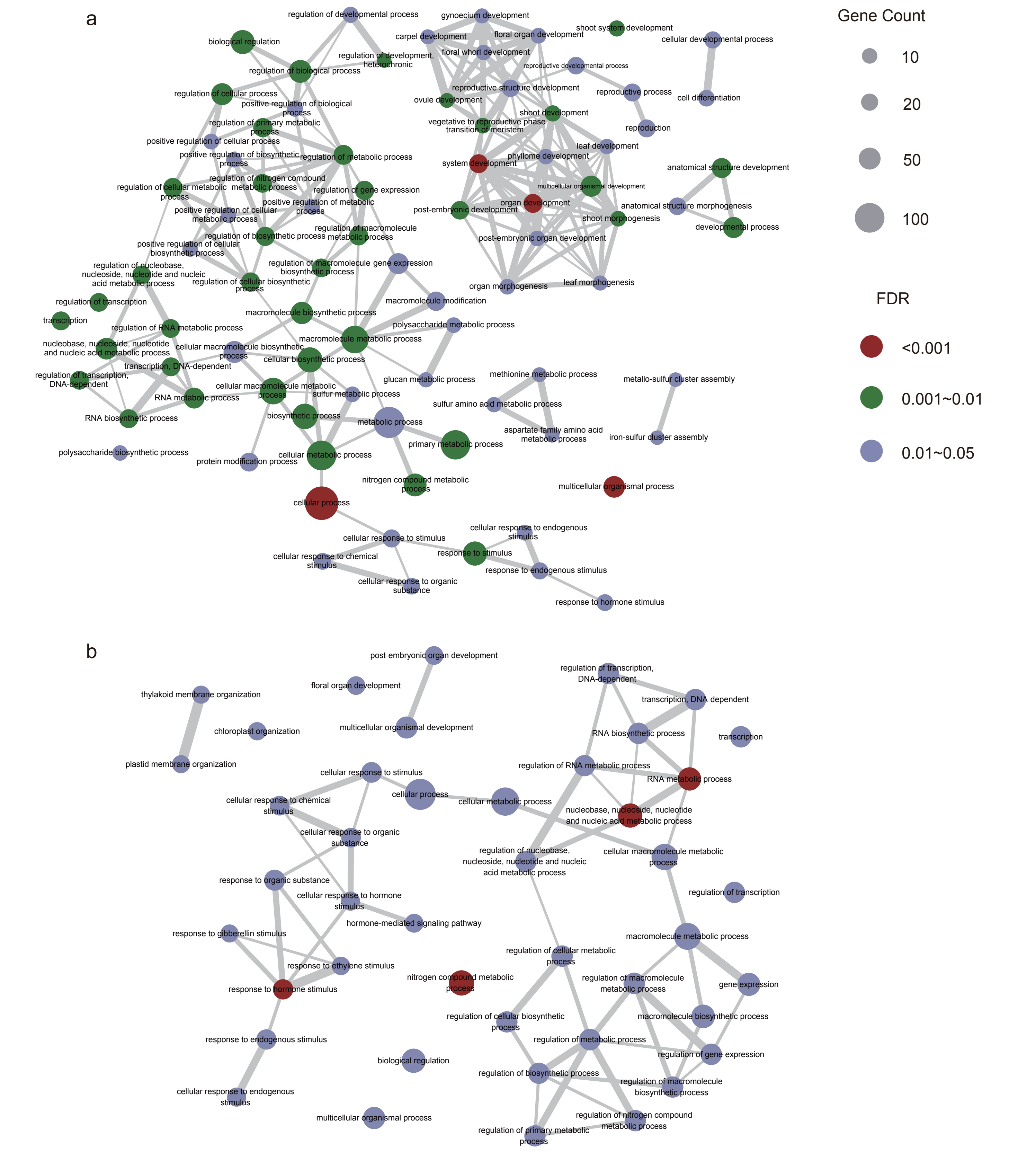
a
Gene Count
10
20
50
100
FDR
<0.001
0.001~0.01
0.01~0.05
regulation of developmental process
gynoecium development
carpel development
floral organ development
shoot system development
cellular developmental process
biological regulation
regulation of development, heterochronic
floral whorl development
regulation of biological process
reproductive developmental process
regulation of cellular process
positive regulation of biological process
reproductive structure development
ovule development
reproductive process
cell differentiation
regulation of primary metabolic process
shoot development
vegetative to reproductive phase transition of meristem
reproduction
positive regulation of cellular process
positive regulation of biosynthetic process
regulation of metabolic process
leaf development
system development
phyllome development
anatomical structure development
regulation of nitrogen compound metabolic process
regulation of cellular metabolic process
regulation of gene expression
multicellular organismal development
positive regulation of metabolic process
organ development
post-embryonic development
anatomical structure morphogenesis
positive regulation of cellular metabolic process
shoot morphogenesis
developmental process
regulation of biosynthetic process
regulation of macromolecule metabolic process
post-embryonic organ development
positive regulation of cellular biosynthetic process
regulation of nucleobase, nucleoside, nucleotide and nucleic acid metabolic process
regulation of macromolecule gene expression
regulation of macromolecule biosynthetic process
regulation of cellular biosynthetic process
organ morphogenesis
leaf morphogenesis
regulation of transcription
macromolecule modification
transcription
macromolecule biosynthetic process
regulation of RNA metabolic process
polysaccharide metabolic process
nucleobase, nucleoside, nucleotide and nucleic acid metabolic process
cellular macromolecule biosynthetic process
macromolecule metabolic process
transcription, DNA-dependent
cellular biosynthetic process
regulation of transcription, DNA-dependent
glucan metabolic process
methionine metabolic process
metallo-sulfur cluster assembly
cellular macromolecule metabolic process
RNA metabolic process
sulfur metabolic process
sulfur amino acid metabolic process
RNA biosynthetic process
biosynthetic process
metabolic process
aspartate family amino acid metabolic process
iron-sulfur cluster assembly
primary metabolic process
polysaccharide biosynthetic process
cellular metabolic process
protein modification process
nitrogen compound metabolic process
multicellular organismal process
cellular process
cellular response to endogenous stimulus
cellular response to stimulus
response to stimulus
cellular response to chemical stimulus
response to endogenous stimulus
cellular response to organic substance
response to hormone stimulus
b
post-embryonic organ development
regulation of transcription, DNA-dependent
floral organ development
thylakoid membrane organization
transcription, DNA-dependent
chloroplast organization
multicellular organismal development
RNA biosynthetic process
transcription
plastid membrane organization
cellular response to stimulus
regulation of RNA metabolic process
RNA metabolic process
cellular process
cellular response to chemical stimulus
cellular metabolic process
nucleobase, nucleoside, nucleotide and nucleic acid metabolic process
cellular response to organic substance
regulation of nucleobase, nucleoside, nucleotide and nucleic acid metabolic process
cellular macromolecule metabolic process
response to organic substance
cellular response to hormone stimulus
regulation of transcription
hormone-mediated signaling pathway
response to gibberellin stimulus
macromolecule metabolic process
regulation of cellular metabolic process
response to ethylene stimulus
nitrogen compound metabolic process
regulation of macromolecule metabolic process
gene expression
response to hormone stimulus
regulation of cellular biosynthetic process
macromolecule biosynthetic process
response to endogenous stimulus
regulation of metabolic process
biological regulation
regulation of gene expression
regulation of biosynthetic process
regulation of macromolecule biosynthetic process
cellular response to endogenous stimulus
regulation of nitrogen compound metabolic process
multicellular organismal process
regulation of primary metabolic process
